# Regulation of heme oxygenase-1 mRNA deadenylation and turnover in NIH3T3 cells by nitrosative or alkylation stress

**DOI:** 10.1186/1471-2199-8-116

**Published:** 2007-12-20

**Authors:** Veronica Leautaud, Bruce Demple

**Affiliations:** 1 Department of Genetics and Complex Diseases, Harvard School of Public Health, 665 Huntington Avenue, Boston, MA 02115, USA; 2 Rice University, Department of Bioengineering MS-142, 6100 Main St, Houston, TX 77005, USA

## Abstract

**Background:**

Heme oxygenase-1 (HO-1) catalizes heme degradation, and is considered one of the most sensitive indicators of cellular stress. Previous work in human fibroblasts has shown that HO-1 expression is induced by NO, and that transcriptional induction is only partially responsible; instead, the HO-1 mRNA half-life is substantially increased in response to NO. The mechanism of this stabilization remains unknown.

**Results:**

In NIH3T3 murine fibroblasts, NO exposure increased the half-life of the HO-1 transcript from ~1.6 h to 11 h, while treatments with CdCl_2_, NaAsO_2 _or H_2_O_2 _increased the half-life only up to 5 h. Although poly(A) tail shortening can be rate-limiting in mRNA degradation, the HO-1 mRNA deadenylation rate in NO-treated cells was ~65% of that in untreated controls. In untreated cells, HO-1 poly(A) removal proceeded until 30–50 nt remained, followed by rapid mRNA decay. In NO-treated cells, HO-1 deadenylation stopped with the mRNA retaining poly(A) tails 30–50 nt long. We hypothesize that NO treatment stops poly(A) tail shortening at the critical 30- to 50-nt length. This is not a general mechanism for the post-transcriptional regulation of HO-1 mRNA. Methyl methane sulfonate also stabilized HO-1 mRNA, but that was associated with an 8-fold decrease in the deadenylation rate compared to that of untreated cells. Another HO-1 inducer, CdCl_2_, caused a strong increase in the mRNA level without affecting the HO-1 mRNA half-life.

**Conclusion:**

The regulation of HO-1 mRNA levels in response to cellular stress can be induced by transcriptional and different post-transcriptional events that act independently, and vary depending on the stress inducer. While NO appears to stabilize HO-1 mRNA by preventing the final steps of deadenylation, methyl methane sulfonate achieves stabilization through the regulation of earlier stages of deadenylation.

## Background

Nitric oxide is a major messenger molecule with a broad array of physiological functions, which include the ability to promote cell survival during conditions of serum starvation and oxidative stress [1,2]. At the same time, NO is a key effector of the cell-mediated immune response, acting as a cytotoxic agent against invading pathogens [3], as well as having the potential to cause DNA damage and cell death in mammalian cells [4,5]. Such diverse and sometimes opposing roles can be better understood by considering the complex chemistry of NO. NO can react directly with iron in heme and iron-sulfur proteins or, it can react with superoxide to form highly oxidizing peroxynitrite. NO can also react with oxygen to generate higher nitrogen oxides (N_2_O_3_, N_2_O_4_) in which NO is oxidized to the nitrosonium ion (NO^+^), the NO redox form responsible for its reactions with thiols [6,7].

In this way NO elicits both nitrosative and oxidative stress, conditions that result in heme oxygenase-1 (HO-1) up-regulation [8-10]. HO-1 catalyzes heme degradation, and is induced by a variety of stress inducers, such as cadmium chloride (CdCl_2_), sodium arsenite (NaAsO_2_) and hydrogen peroxide (H_2_O_2_), an effect that is consistent with the role of HO-1 as a protective enzyme [11-13]. The relevance of HO-1 induction in response to NO has become apparent from studies with HO-1 knockout mice and tissues, which show increased sensitivity to endotoxin (a known inducer of NO synthase) [14]. Furthermore, motor neurons respond to sub-lethal fluxes of NO by activating defense mechanisms that allow them to survive a subsequent exposure to higher doses of NO. Under these conditions, HO-1 is induced and its activity is required for this adaptive resistance to NO [15-17].

HO-1 mRNA levels are also increased in response to NO in human IMR90 lung fibroblasts, and transcriptional induction is only partially responsible. Instead, the HO-1 mRNA half-life is substantially increased from ~2 h in controls to >10 h in NO-treated cells [18,19]. Thus, NO can regulate the HO-1 mRNA decay process and in this way contributes to the up-regulation of HO-1 expression.

The decay of mRNA is a tightly controlled process dependent on a core machinery for poly(A) tail shortening and 5'-decapping, as well as a 5'→3' nuclease, and the exosome responsible for 3'→5'decay [20,21]. In addition to these factors, transcript-specific sequence elements and trans-factors that bind them regulate the decay process [22]. In general, poly(A) tail shortening (deadenylation) is the rate-limiting step in this process, since it is required for both 5'→3' and 3'→5' decay [23], and for several transcripts decapping does not normally occur until the poly(A) tail is sufficiently shortened [24].

Consistent with poly(A) tail shortening as a rate-limiting step in mRNA decay, this step is targeted for regulation in at least two other pathways: nonsense-mediated decay (NMD) and decay of mRNA containing AU-rich sequence elements (AREs). During deadenylation-dependent nonsense-mediated decay, the presence of a premature termination codon results in an increased rate of poly(A) tail shortening and subsequent decay of the aberrant transcripts [25,26]. Shortening of the poly(A) tail is also relevant for the decay of transcripts such as granulocyte/monocyte colony stimulating factor, c-*fos *and c-*myc*, whose half-life is regulated by AREs [27]. These sequence elements can act in *cis *to increase the rate of poly(A) tail shortening and the overall decay of a stable β-globin transcript [28,29]. AREs mediate responses to cellular stresses and pro-inflammatory stimuli, as evidenced by the regulation of ARE-dependent deadenylation by p38 mitogen-activated protein kinase [30]. ARE-binding proteins such as AUF1 and HuR have been shown to regulate mRNA half-life in response to genotoxic and oxidative stress [31,32], and ARE-binding proteins that promote or inhibit poly(A) tail shortening, such as tristetraprolin or HuD, control the half-life of mRNA and are regulated by growth factors, endotoxin, pro-inflammatory cytokines and other extracellular stimuli [33-36]. Thus mRNA poly(A) tail shortening can be a key step in an mRNA decay pathway that is tightly regulated in response to extracellular stimuli.

In this study, we have begun to dissect the mechanistic pathway of HO-1 mRNA turnover and the effects of NO. We examined the generality of HO-1 post-transcriptional regulation in response to other sources of cellular stress. We have also identified a novel regulatory mechanism in which the initial rate of HO-1 mRNA poly(A) tail shortening in response to NO remains largely unaffected, with stabilization achieved by preventing the final deadenylation steps or decapping. These results provide insights into the mechanism of post-transcriptional regulation and the role of deadenylation during cellular stress responses.

## Results

### NO-mediated stabilization of HO-1 mRNA in NIH3T3 cells

Previous studies in human IMR90 fibroblasts showed that HO-1 mRNA is stabilized in response to NO [18,19]. However, because these primary cells senesce in culture and posed experimental limitations, as an alternative we tested mouse NIH3T3 fibroblasts for NO-induced stabilization of the HO-1 message. To provide controlled NO exposure in culture, we used the diazeniumdiolate NO donors SPER/NO and DETA/NO, which release two molecules of NO per donor molecule at a steady and reproducible rate [37]. We used SPER/NO for a 1-h NO exposure (nominal half-life 45 min), or DETA/NO (nominal half-life 20 h) for longer exposures to NO.

**Figure 1:**
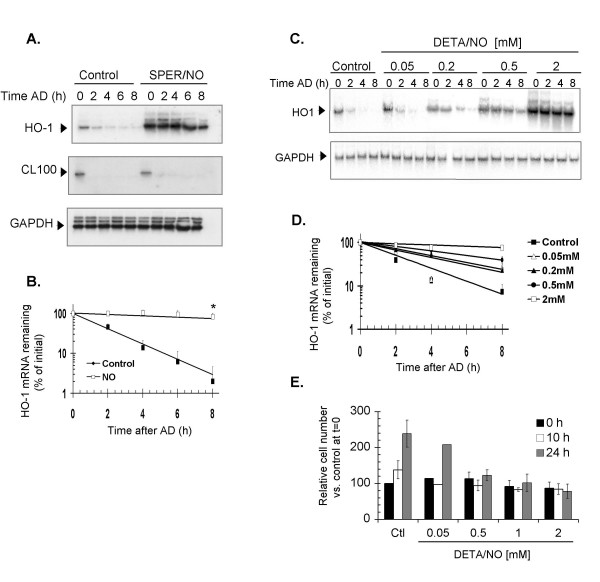
**Effect of NO exposure on HO-1 mRNA induction and stabilization**. **A**. NIH3T3 cells were treated as controls or with an acute exposure to 0.5 mM SPER/NO for 1 h. AD was added to block transcription and total RNA was extracted at the indicated times. HO-1 mRNA expression was monitored by northern blotting and normalized to GAPDH expression. The same blot was also probed for the expression of unstable CL100 mRNA.**B**. Quantification shows the fitted decay lines, calculated from three independent experiments. Data points show the mean and standard errors. * p < 0.05 as compared the HO-1 mRNA remaining (% of initial) in untreated controls at the corresponding times. **C**. NIH3T3 cells were treated with the indicated concentrations of DETA/NO for 6 h prior to the addition of AD, and the percentage of HO-1 mRNA remaining was quantified at the indicated time points.**D**. Quantification shows the fitted decay lines, calculated from two independent experiments. Data points show the mean and standard errors. **E**. In parallel experiments, cells were trypsinized immediately after the treatment or at 8 h and 24 h after treatment, followed by trypan blue staining. To estimate viability, the values were normalized to 100% at time 0 h in untreated controls. Quantification in panel E shows the mean and standard error of three independent experiments, except for 0.5 mM dose, which corresponds to one experiment. ** p < 0.01, * p < 0.05 as compared to untreated controls at the corresponding times.

NIH3T3 cells were treated with 0.5 mM SPER/NO for 1 h, after which transcription was blocked by adding 10 μg/ml of AD. In untreated controls, HO-1 mRNA decayed with a 1.6 h half-life. Upon treatment with SPER/NO, HO-1 mRNA was stabilized with a half-life of 11 h (Fig. 1A, B). To examine the specificity of this post-transcriptional regulation by NO, we measured in the same samples the half-life of the unstable, stress-inducible transcript encoding CL100 phosphatase [38]. Treatment with SPER/NO did not stabilize CL100 mRNA (Fig. 1A), suggesting that the effect on HO-1 mRNA is not due to a general inhibition of NO on cellular mRNA turnover. Moreover, recent microarray results from this laboratory indicate that the stability of the vast majority of mRNAs in NIH3T3 or IMR90 cells is unaffected by NO (Rabinovic et al., manuscript in preparation).

HO-1 mRNA induction experiments were also carried out using a prolonged exposure to NO, in which NIH3T3 cells were monitored in parallel for cell viability by trypan blue exclusion. Increasing doses of DETA/NO resulted in increased HO-1 mRNA stability, with the half-life ranging from 1.6 h in controls, to 4.7 h, 7 h, and 11 h after DETA/NO treatments of 0.2 mM, 0.5 mM and 2 mM, respectively (Fig. 1C, D). There was also a negative correlation between the DETA/NO level and cell proliferation during 24 h after the treatment (Fig. 1E). Although NO did not seem to affect the viable cell number immediately after the treatment and for up to 10 h, the interval during which mRNA stability was assessed, by 24 h post-exposure an effect on cell proliferation was apparent. The untreated cell population doubled in 24 h, as was true for the cells exposed to 0.05 mM DETA/NO. However, at higher DETA/NO levels, cell proliferation was prevented (Fig. 1E). Note that there was no loss in cell viability immediately after NO treatment with up to 1 mM DETA/NO, and only a small decrease in viability in cells treated with 2 mM DETA/NO (Fig. 1E), conditions under which the HO-1 mRNA was strongly stabilized (Fig. 1C, D). These effects of DETA/NO on HO-1 mRNA stabilization and cell proliferation, were similarly produced by treatment with S-nitroso-L-glutathione (Additional File 1). Thus NIH3T3 cells are a suitable model to further characterize the mechanism of HO-1 mRNA stabilization by NO.

### Effect of CdCl_2_, NaAsO_2 _and H_2_O_2 _on HO-1 mRNA induction and stabilization

To determine whether the post-transcriptional regulation of HO-1 mRNA was specific to NO or part of a general stress response, and to inform the chemistry of this signaling pathway, we tested several compounds chemically unrelated to NO for their effect on HO-1 mRNA stability. The molecular targets of these compounds overlap partially with those of NO. In particular, the reactions of NO with thiols to form nitrosothiols [39], and the eventual depletion of intracellular glutathione [40,41], prompted us to investigate the oxidant H_2_O_2_, and the thiol-reactive compounds CdCl_2 _and NaAsO_2_, which can alter the redox equilibrium [42,43].

**Figure 2:**
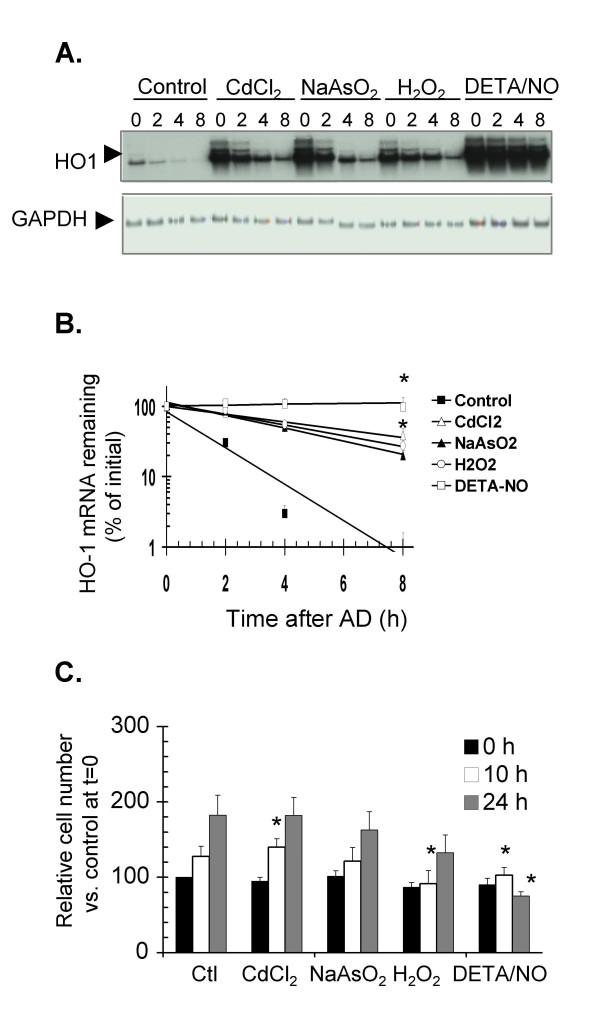
**HO-1 mRNA induction and stabilization in response to CdCl_2_, NaAsO_2 _or H_2_O_2_**. **A**. Cells were treated with 2 mM DETA/NO, 25 μM CdCl_2_, 10 μM NaAsO_2_, 250 μM H_2_O_2 _or 2 mM DETA/NO for 6 h, followed by the addition of AD to monitor HO-1 decay over time. HO-1 mRNA was detected by northern blotting and normalized to GAPDH loading controls.**B**. For each treatment in panel A, the fitted decay lines were calculated using data from three independent experiments and plotted. Data points show the mean and standard errors. * p < 0.05 as compared to HO-1 mRNA expression in control samples at the corresponding times. **C**. The same doses as for panel A were used to determine the effect of these treatments on NIH3T3 cell number, expressed as the percentage of trypan blue-negative cells compared to 100% at time 0 h for untreated controls. Quantification shows the mean and standard error of three independent experiments. * p < 0.05 as compared to untreated controls at the corresponding times.

The contribution of post-transcriptional events in regulating expression of the HO-1 mRNA was determined for different concentrations of CdCl_2_, NaAsO_2 _and H_2_O_2 _in a 6-h exposure. All three agents, at concentrations that induced HO-1 mRNA to steady-state levels comparable to those produced by NO (data not shown), had an effect on HO-1 mRNA stability, but in all cases less than the effect observed for NO treatment (Fig. 2A, B). Under our conditions, CdCl_2_, NaAsO_2 _and H_2_O_2_increased the half-life of HO-1 mRNA to 5–6 h, while a treatment with 2 mM DETA/NO resulted in an HO-1 mRNA half-life of 11 h (Fig. 2A, B). Thus, other sources of cellular stress can affect HO-1 mRNA stability. At the concentrations used, CdCl_2_, NaAsO_2 _and H_2_O_2 _did not prevent cell proliferation throughout a 24-h time course after the treatments, in contrast to the decreased cell proliferation and viability observed in response to NO. (Fig. 2C).

### Effect of methyl methane sulfonate on HO-1 mRNA stability

**Figure 3:**
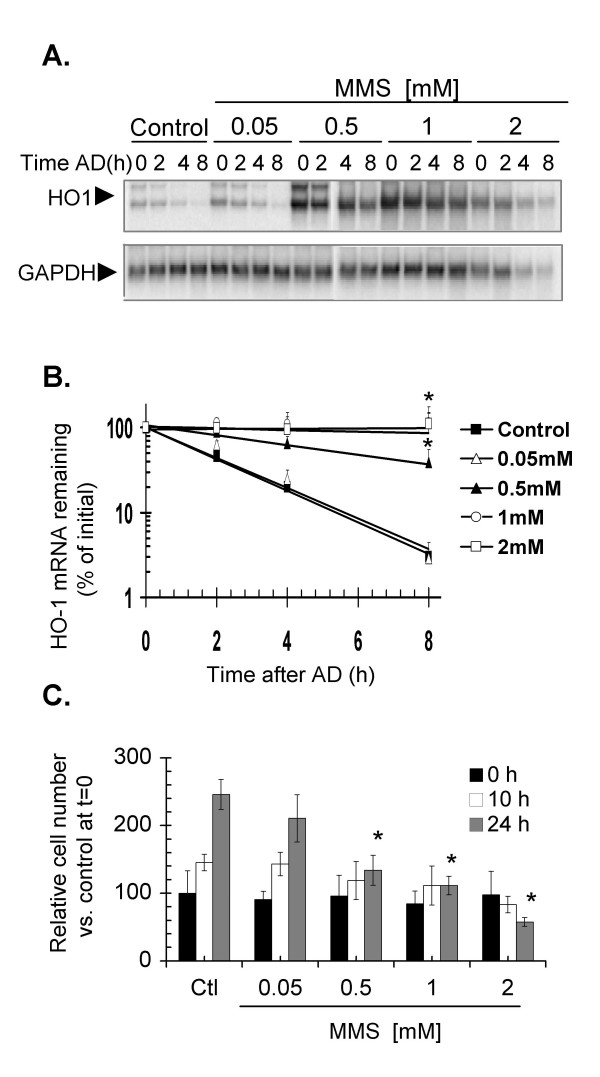
**Effect of increasing concentrations of MMS on HO-1 mRNA turnover and NIH3T3 cell proliferation**. **A**. Cells were treated with the indicated concentrations of MMS for 6 h, followed by the addition of AD and RNA extraction at the indicated time points. HO-1 mRNA was detected by northern blotting and normalized to GAPDH loading controls.**B**. For each concentration in panel A, the fitted decay lines were calculated using data from three independent experiments and plotted. Data points show the mean and standard errors. **C**. In parallel experiments viable cells (trypan blue-negative) were counted immediately after the treatment (time 0 h) and at 8 h and 24 h after treatment. The percentage of viable cells shown is relative to 100% cell number at time 0 h in untreated controls. The graph shows the mean and standard error of three independent experiments. * p < 0.05 as compared to untreated controls at the corresponding times.

To address the possibility that inhibition of cell proliferation stabilizes HO-1 mRNA, we treated NIH3T3 cells with methyl methane sulfonate (MMS), a compound that alkylates DNA and other cellular targets in a non-oxidative fashion. Treatment with MMS affected both cell viability and HO-1 mRNA stability (Fig. 3). At 0.5 mM MMS, HO-1 mRNA was partly stabilized, and treatment with 1 mM MMS resulted in an HO-1 mRNA half-life of >12 h, comparable to that attained with NO-treatment. However, the stabilizing MMS treatments were accompanied by strong inhibition of cell proliferation and reduced viability 24 h after the exposure, and in the 2 mM treatment, decreased GAPDH mRNA levels (Fig. 3C).

### HO-1 mRNA stabilization is not due to the use of an alternative transcriptional start site

For most mRNAs, decay begins with shortening of the 3' poly(A) tail followed by 5' decapping and then rapid digestion of the body of the message. In some cases, stability is regulated through the use of alternative polyadenylation sites [44,45], or through alternative transcriptional start sites to give different 5' untranslated regions (UTRs) [46,47]. We have closely examined the short 93-nt 3'UTR of HO-1 for known polyadenylation motifs [45], and have only found a single polyadenylation motif, which suggests that HO-1 mRNA is not regulated through an alternative polyadenylation site in this region. Additional potential polyadenylation motifs also seem to be absent from the murine HO-1 gene. We therefore performed primer extension assays to determine the transcriptional start site of HO-1 mRNA in control and NO-treated cells (Additional File 2). The HO-1 transcripts from both the control NIH3T3 and the NO-treated cells yielded main products of indistinguishable mobility, which corresponded to the reported mouse HO-1 transcriptional start site [48]. Primer extension products from cells treated with CdCl_2 _and MMS were also similar to products from untreated cells, and again corresponded to the reported transcriptional start site (data not shown). Thus, HO-1 mRNA induction by these agents was not associated with the use of an alternative transcriptional start site.

### NO does not prevent deadenylation but affects the deadenylation rate

Shortening of the 3' poly(A) tail (deadenylation) is often rate-limiting in mRNA decay, and we therefore developed a deadenylation assay specific for endogenous HO-1 mRNA. DNA oligonucleotides were used to target RNase H cleavage prior to separation of the fragments by gel electrophoresis and detection by northern blotting [28]. The length of the poly(A) tail in HO-1 mRNA was estimated by comparison against size markers, including a fully deadenylated HO-1 fragment generated *in vitro*.

**Figure 4:**
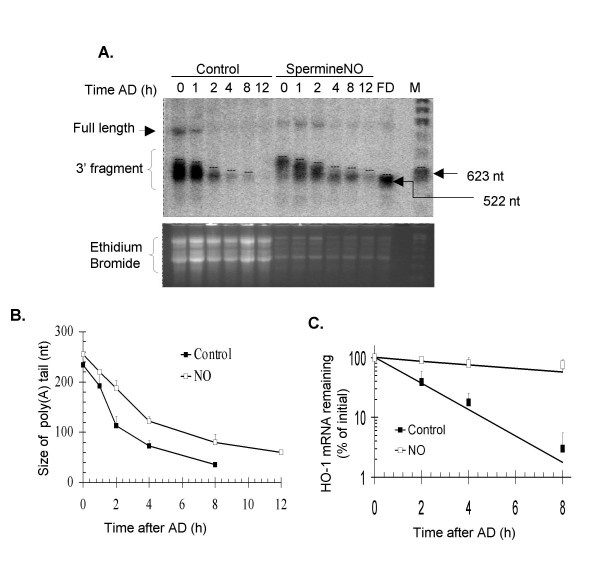
**Effect of NO on HO-1 mRNA deadenylation and stability**. **A**. NIH3T3 cells were treated as controls or with NO, and total RNA samples were assayed for HO-1 mRNA deadenylation. To compensate for the differences in the starting amount of HO-1 mRNA between control and NO-treated cells where HO-1 mRNA is induced immediately after the SPER/NO treatment, 35 μg and 6 μg of total mRNA were used in a cleavage reaction from untreated controls and NO-treated samples, respectively. Residual full-length HO-1 mRNA and the 3' end fragment carrying the poly(A) tail are indicated. FD represents HO-1 mRNA that was fully deadenylated *in vitro*. **B**. The upper boundary of the mRNA was set at a position below which 95–97% of the total HO-1 mRNA band area was included. The size of the poly(A) tail was estimated by comparing the migration of the upper bound to that of the FD (522-nt) and 623-nt size markers. **C**. A fraction of the samples was used without further modification to assess the half-life of HO-1 mRNA by northern blotting. Quantification in panels B and C shows the fitted decay lines calculated from two independent experiments. Data points show the mean and standard errors.

NIH3T3 cells were treated with 0.5 mM SPER/NO for 1 h, followed by the addition of AD and sampling at various times thereafter. Part of each sample was used without further modification to assess the half-life of HO-1 mRNA (Fig. 4C), and the rest of the sample was used in a deadenylation assay. Upon oligo-directed cleavage, as seen in Fig. 4A, two distinct HO-1 mRNA populations were detected in the samples from NO-treated cells: a steady-state population that had undergone different extents of poly(A) tail shortening, and newly transcribed HO-1 mRNA that had the longest poly(A) tails of ~250-nt (Fig. 4A, compare control and NO-treated samples at 0 h). The latter mRNA population resembles the product expected from a transcriptional pulse [27], consistent with HO-1 induction by NO including both transcriptional (during the NO exposure) and post-transcriptional components [19]. In this regard, it is important to note that because our experiments have the advantage of analyzing the endogenous HO-1 mRNA rather than an inducible transcript, the starting point of our experiments represents the steady state population of mRNAs that have undergone different extents of decay. Thus, evaluating the average intensities in our deadenylation assays would neglect to consider the actual rate of shortening of the longer poly(A) tails. Therefore, we marked the position below which 95–98% of the signal remained, and considered this upper 2–5% boundary to correspond to the mRNAs with the longest poly(A) tails, and the only part of the signal where we could accurately monitor poly(A) tail shortening (see dotted lines in each lane of Fig. 4A). This analysis showed that poly(A) shortening of HO-1 mRNA occurred in both the control and the NO-treated samples (Fig. 4B). From these results, we estimated that, during the first 8 h of the time course, the initial rate of deadenylation was about 1.5-fold slower in the NO-treated samples (1.3-nt per min) than in the controls (1.9-nt per min).

Fully deadenylated HO-1 mRNA was not detected in either the control or the NO-treated case. The smallest detectable fragments corresponded to a poly(A) tail of 30–50 residues, which suggests that rapid decay occurs if the poly(A) tail is shortened beyond this point. In NO-treated cells, poly(A) tail shortening up to 30–50 nt occurred in spite of the increased HO-1 mRNA half-life. Importantly, in contrast to controls, these shorter decay intermediates were readily detected at later time points in the NO-treated samples (Fig. 4A, compare control and NO-treated samples at 8 h and 12 h).

### MMS treatment stabilizes HO-1 mRNA by affecting the deadenylation rate

**Figure 5:**
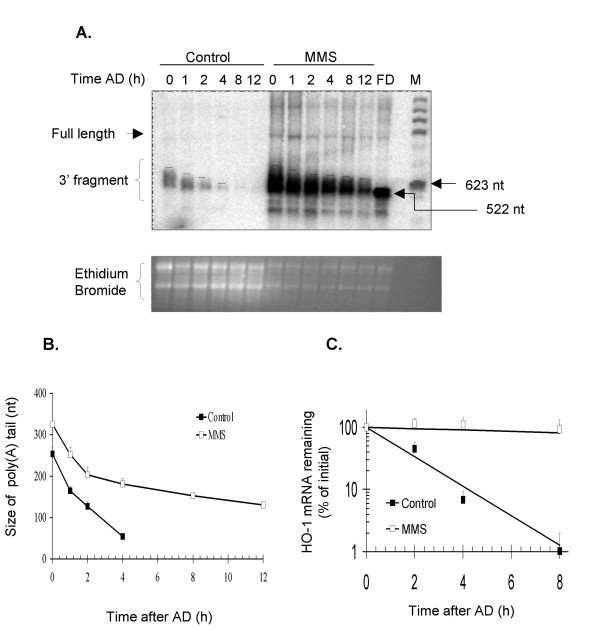
**Effect of MMS on HO-1 mRNA deadenylation and stability**. **A**. NIH3T3 cells were treated as controls or with 1 mM MMS for 6 h, and total RNA samples (20 μg and 7.5 μg, respectively) were assayed for HO-1 mRNA deadenylation. Residual full-length mRNA and the 3' end fragment carrying the poly(A) tail are indicated. FD represents a fully deadenylated 3' fragment of HO-1 mRNA. **B**. The size of the poly(A) tail was estimated as for Fig. 5. **C**. A fraction of these samples was used without further modification to assess the half-life of HO-1 mRNA. Quantification in panels B and C shows the fitted decay lines calculated from two independent experiments. Data points show the mean and standard errors.

To determine whether altered deadenylation of HO-1 mRNA is a general mechanism for its post-transcriptional regulation, we analyzed this process in MMS-treated cells. We treated NIH3T3 cells with 1 mM MMS for 6 h, and followed this treatment with the addition of AD to the medium to monitor the deadenylation and decay of HO-1 mRNA through a 12-h time course. HO-1 mRNA expression was induced 10-fold by the MMS treatment and its mRNA was stabilized, with the half-life increased from 2 h in control samples to >12 h in MMS-treated cells (Fig. 5). In control and MMS-treated samples both polyadenylated intermediates and partially deadenylated HO-1 mRNA were detected immediately after the addition of AD (Fig. 5A). In control samples, HO-1 mRNA underwent significant poly(A) tail shortening and decay by 4 h (Fig. 5A, C), whereas treatment with MMS significantly decreased the HO-1 mRNA deadenylation rate. Although during the first 2 h of the AD time course the rate was only 1.5-fold slower than in controls, the effect of MMS on the HO-1 mRNA deadenylation rate was more pronounced after 2 h: after initial shortening to a poly(A) size of ~200-nt, the deadenylation rate in the MMS-treated samples was ~8-fold slower than in controls.

### Transcriptional up-regulation does not result in HO-1 mRNA stabilization

Previous experiments in human IMR90 fibroblasts suggested that transcription inhibition may partially affect the stabilization of HO-1 mRNA by NO, decreasing the mRNA half-life from 10 h to 6 h [18]. Since transcription is tightly coupled to mRNA splicing, polyadenylation, capping, export, and stability [49,50], we decided to address the effect of transcription on HO-1 mRNA stability.

**Figure 6:**
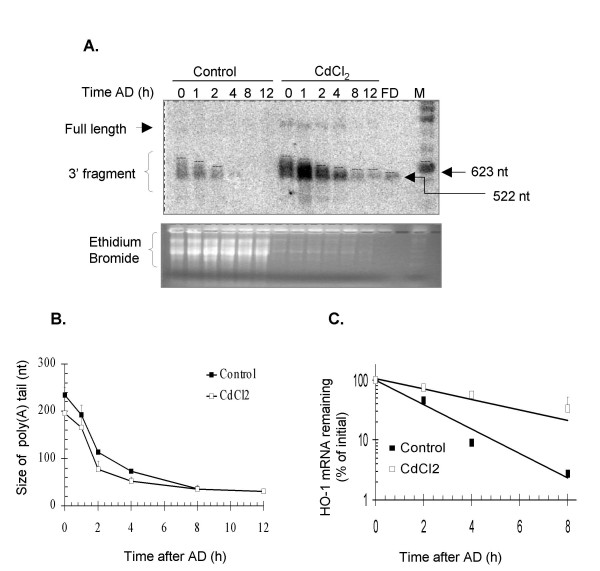
**Effect of CdCl_2 _on HO-1 mRNA deadenylation and stability**. **A**. NIH3T3 cells were treated as controls or with 2.5 μM CdCl_2 _for 6 h, and total RNA was assayed for HO-1 mRNA deadenylation. Residual full-length mRNA and the 3' end fragment carrying the poly(A) tail are indicated. FD represents the fully deadenylated 3' fragment of HO-1 mRNA. **B**. The size of the poly(A) tail was estimated as for Fig. 5. **C**. A fraction of the samples was used without further modification to assess the half-life of HO-1 mRNA. Quantification in panels B and C shows the fitted decay lines calculated from two independent experiments. Data points show the mean and standard errors.

To this end, NIH3T3 cells were treated with CdCl_2 _under conditions that resulted in a 4-fold HO-1 mRNA induction, similar to that of NO. The CdCl_2 _treatment increased the HO-1 mRNA half-life only modestly to ~4 h (Fig. 2B and 6C). As seen in Fig. 6A, HO-1 mRNA underwent deadenylation in CdCl_2_-treated cells with an initial rate similar to that estimated for the untreated controls (1.7-nt per min in CdCl_2 _samples vs. 1.9-nt per min in controls). These results suggest that post-transcriptional events contribute only marginally to the HO-1mRNA up-regulation in response to CdCl_2_.

## Discussion

In this study we established that NIH3T3 mouse fibroblasts showed an increase in HO-1 mRNA steady-state levels in response to acute exposure to NO released from SPER/NO. This increase also correlated with a significant increase in the HO-1 mRNA half-life, which was also observed in response to a prolonged NO exposure using a slow-releasing diazeniumdiolate, DETA/NO or S-nitrosoglutathione. These observations are in agreement with published reports showing strong HO-1 mRNA induction in response to NO in other cell types [8-10,16,17] and with its induction and stabilization in human IMR90 fibroblasts [18,19] and rat aortic smooth muscle cells in response to various sources of NO [10]. On the other hand, we have not been able to demonstrate NO-mediated stabilization of the HO-1 message in HeLa or HepG2 cells (unpublished data), which suggests that the mechanism may be limited to certain cell types.

Treatment with NO, at concentrations that increased HO-1 mRNA stability, prevented cell proliferation during the 24 h after exposure. This observation is in agreement with the ability of NO to cause cell cycle arrest [51,52]. It also parallels reports where exposure to DETA/NO prevented NIH3T3 cell proliferation in a p53-dependent manner, causing the induction of several growth-arrest genes: p21, GADD45 and Bax [53]. The induction and stabilization of HO-1 mRNA under stress conditions that compromise cell growth is consistent with its proposed role in mediating cell survival during nitrosative and oxidative stress [14,16,17].

Also in agreement with this role is the induction and stabilization of HO-1 in response to CdCl_2_, NaAsO_2 _and H_2_O_2_. These agents, although chemically unrelated to NO, have overlapping effects on cellular physiology through the depletion of cellular glutathione, which shifts the redox equilibrium in the cell [42,43], and can also affect the cell cycle and viability [54-58]. It is important to note that, in our study, we used exposures to CdCl_2_, NaAsO_2 _and H_2_O_2 _similar to those previously reported to induce HO-1expression [11-13]. These concentrations were lower than those reported to affect the cell cycle and, in agreement with this, failed to block cell proliferation during 24 h after the exposure. It is then possible that higher concentrations of these agents are required to achieve the degree of HO-1 mRNA stabilization seen with NO. In the case of CdCl_2_, concentrations of 75 μM and higher were needed to stabilize HO-1 mRNA, but under these conditions, stability was accompanied by massive ribosomal and messenger RNA degradation (data not shown). The increase in the HO-1 mRNA half-life observed in response to the non-NO agents, at concentrations that resulted in a similar HO-1 mRNA induction to that elicited by NO, shows that other sources of cellular stress may regulate HO-1 gene expression post-transcriptionally, and that HO-1 mRNA stabilization is not exclusive to NO. Overall, however, the most dramatic increase in HO-1 mRNA half-life was consistently observed with NO. Therefore it is possible that, in addition to thiol-dependent reactions and oxidative stress, other NO-specific reactions might mediate the post-transcriptional regulation of HO-1.

Because NO treatments that resulted in HO-1 mRNA stabilization were also associated with a decrease in cell proliferation, we assessed HO-1 mRNA stability in response to MMS, a cytotoxic alkylating agent. In agreement with published reports [59,60], increasing concentrations of MMS caused a decrease in the number of viable cells over time. Interestingly, although chemically unrelated to NO, MMS exerted significant stabilization of the HO-1 mRNA, expanding the list of known HO-1 inducers. In this respect it is noteworthy that MMS can activate mitogen-activated protein kinase (MAPK) pathways in a DNA damage-independent manner, modulated by the intracellular level of glutathione [61], perhaps through the ability of MMS to alkylate sulfhydryl groups. Therefore, as HO-1 mRNA induction has been reported to depend on the activation of MAPK pathways in some cell types [11,12,57,62], and HO-1 up-regulation is modulated by the intracellular glutathione status (reviewed in [63]), it is possible that MMS and NO regulate HO-1mRNA induction and stabilization through overlapping mechanisms.

To begin to address the mechanism of post-transcriptional regulation of HO-1 in NIH3T3 cells, and because no evidence for alternative polyadenylation sites within the HO-1 gene was found, we tested whether HO-1 induction by NO is accompanied by the use of an alternative transcriptional start site, which could in turn result in differences in the 5' untranslated region of HO-1 mRNA and affect its stability. Such an effect has been reported for unstable c-*myc *mRNA and IGFII mRNA, which can be transcribed from different promoters, and whose stability is regulated through the use of alternative transcriptional start sites [46,47]. Unlike these cases, the HO-1 transcriptional start site was not affected by any of the stabilizing treatments (NO exposure or MMS), consistent with other data showing that HO-1 mRNA post-transcriptional regulation by NO can occur independently of transcription [18].

We also monitored the effect of NO and other stress inducers on HO-1 mRNA deadenylation, since deadenylation has been described as the rate-limiting step in mRNA decay [23-27]. In stabilizing HO-1 mRNA, NO had only a small effect on the initial rate of poly(A) tail shortening, acting instead to prevent the complete deadenylation of HO-1 mRNA past a 30–50-nt critical poly(A) tail length. A minimum poly(A) tail past which degradation occurs has been previously described for globin and histone mRNAs injected in Xenopus laevis extracts (reviewed in [64]), although such a critical length is not applicable to all mRNAs, because in this system the half-life of interferon mRNAs was independent of the length of their poly(A) tail [65]. In this regard, it is interesting to note that, a significant amount of the HO-1 mRNA from NO-treated samples remained sufficiently polyadenylated to be retained on oligo(dT) Sepharose 8 h after treatment (Additional File 3: Recovery of polyadenylated (A_>30_) HO-1 mRNA from NO-treated cells). An alternative possibility is that NO acts to block decapping, or the activity of endonucleases that might be activated when the poly(A) tail is significantly shortened. This mechanism of stabilization of HO-1 mRNA is in contrast to the mRNA post-transcriptional regulation described for NMD and ARE-mediated decay, where the half-life of a transcript containing a premature termination codon or an ARE is regulated through changes in its deadenylation rate [25-27]. In our experiments, we estimated that the >5-fold increase in the HO-1 half-life that occurs in response to NO is unlikely to be the result of differences in the HO-1 deadenylation rate, because the poly(A) tail of HO-1 mRNA in NO-treated cells was shortened with a rate that was only 1.5-fold slower than that of control cells.

Our experiments with MMS show that a different form of cellular stress can also stabilize HO-1, but by a different mechanism that involves a dramatic change in the rate of shortening the poly(A) tail from ~200-nt down. We have also demonstrated that, in spite of strong transcriptional induction of HO-1 in response to CdCl_2_, this agent did not strongly affect the rate of poly(A) tail shortening and had only a small effect on the HO-1 mRNA half-life. As CdCl_2_-induced transcription did not change the start site, this experiment further supports the conclusion that stabilization occurs independently of HO-1 mRNA transcription, and excludes the possibility that detection of oligoadenylated HO-1 mRNA through the time course is merely a by-product of elevated HO-1 mRNA levels prior to the addition of AD in the treated samples.

Here we have characterized a mechanism for the post-transcriptional regulation of HO-1 mRNA where stabilization can occur essentially independently of the initial rate of poly(A) tail shortening, by preventing the final steps of deadenylation or a subsequent step in decay. Such regulation has been reported for the stabilization of transcripts in an *in vitro *decay assay, where purified ELAV proteins did not affect deadenylation of ARE-containing mRNA, but prevented the decay of deadenylated transcripts [66]. Moreover, the differential regulation for the initial rate of poly(A) tail shortening and the terminal deadenylation rate has been described for the GAP-43 mRNA by HuD, a neuronal ARE-binding protein that can prevent poly(A) tail shortening from transcripts containing a long poly(A) tail (A_150_) [34,36,67]. HuD appears to have no effect on the deadenylation and decay rates of GAP-43 transcripts with short tails (A_30_), perhaps because of lower binding affinity for these transcripts [34] than for transcripts with longer poly(A) tails [34]. In a similar way, an NO-induced stabilizing factor that would preferentially bind certain mRNAs with short poly(A) tails (A_30–50_) would affect only the terminal steps of HO-1 mRNA deadenylation. Indeed, some recent experiments demonstrate NO-inducible binding to the 3' UTR of HO-1 mRNA by cellular proteins (A. Rabinovic et al., manuscript in preparation).

Differential regulation of the rate of poly(A) tail shortening and terminal deadenylation could also be achieved though the differential regulation of cellular deadenylases. To date, the activity of PARN, a major cellular deadenylase, has been well characterized in HeLa S100 extracts [68]. In addition to PARN, two yeast deadenylase activities conserved in higher eukaryotes have been characterized: Ccr4/Caf1 and Pan2/Pan3 [69]. While both activities are required for normal deadenylation in yeast, Pan2/Pan3 stops at the last 20–26-nt, perhaps due to its requirement for poly(A) binding protein (Pab1p) as a cofactor, and only the Ccr4/Caf1 complex can process the last phase of deadenylation [23,70]. The Pan2/Pan3 complex has been identified in HeLa cells, where its activity is also stimulated by polyadenylate-binding protein (PABP) [71]. Thus, regulating the activity of one or the other deadenylase would have distinct effects on the rate of poly(A) shortening and terminal deadenylation, and such a mechanism could account for the differential effects of MMS and NO on the rate of HO-1 mRNA poly(A) tail shortening and terminal deadenylation.

## Conclusion

In this work we have demonstrated nitric oxide-induced stabilization of HO-1 mRNA in NIH3T3 murine fibroblasts. HO-1 mRNA stabilization in NIH3T3 cells can also occur less dramatically in response to H_2_O_2_, CdCl_2 _and NaAsO_2_. The stabilizing effect of NO on HO-1 did not appear to be due to a generalized inhibition of cellular mRNA turnover, nor to the activation of an alternative transcriptional start site.

HO-1 mRNA was deadenylated in both control and NO-treated cells, and only slightly more slowly in the latter. In neither case were fully deadenylated transcripts detected, which suggests that poly(A) shortening proceeds to a critical point, beyond which rapid decay of the entire mRNA ensues. NO appears to prevent the final shortening step, or possibly the subsequent decapping reaction. Such a stabilization mechanism is distinct from the effect on HO-1 mRNA stability of MMS, which significantly decreases the poly(A) shortening rate. A third agent, CdCl_2_, induces HO-1 mRNA transcriptionally without a significant effect on deadenylation, which is therefore mechanistically unlinked to transcription for HO-1.

## Methods

### Reagents

Nitric oxide donors spermine diazeniumdiolate (SPER/NO) and DETA NONOate (DETA/NO) were purchased from Alexis Corporation (San Diego, CA). Methyl methane sulfonate (MMS), hydrogen peroxide, cadmium chloride and actinomycinD (AD) were purchased from the Sigma Chemical Co. (St. Louis, MO). RNase H enzyme, ammonium acetate, glycogen, and RNase inhibitors RNAsin and SuperaseIN were purchased from Ambion (Austin, TX). T4-polynucleotide kinase was purchased from New England Biolabs (Beverly, MA).

### Cell culture and treatments

Mouse NIH3T3 fibroblasts were grown in Dulbecco's Modified Eagle Medium supplemented with 10% (v/v) bovine calf serum, 50 international units/ml penicillin, 50 μg/ml streptomycin and 40 μM glutamine.

To determine the stability of HO-1 mRNA, NIH3T3 fibroblasts were seeded in T-25 flasks at a density of 2.5 × 10^5 ^cells per flask and grown for 2 d before the experiment.

Cells were treated with the NONOates, or either CdCl_2_, NaAsO_2_, H_2_O_2 _or methyl methane sulfonate (MMS) at the concentrations and times indicated in the results. Treatment was stopped by replacing the medium with conditioned medium supplemented with the transcriptional inhibitor AD to a final concentration of 10 μg/ml. Cells were frozen in liquid nitrogen at the indicated times throughout the time course and stored at -80°C until RNA extraction. Conditioned medium is medium in which cells have been cultured for 2 d, and is used throughout these experiments to prevent the transcriptional induction of HO-1 mRNA that occurs in response to fresh medium [19].

For the primer extension assays and each time point in the deadenylation assays, 1 × 10^6 ^cells were seeded in T-75 tissue culture flasks, and grown for 2 d before the experiment. Cells were treated with 0.5 mM SPER/NO for 1 h before the addition of AD, as described above. MMS treatments were done by preparing a 120 mM stock and adding it to the cell medium to reach a 1 mM final concentration. CdCl_2 _treatments were done by preparing a 5 mM stock solution and adding enough to the cell media to reach a 2.5 μM final concentration. Both the MMS and the CdCl_2 _treatments were carried out for 6 h before replacing the media with conditioned medium and AD.

### Cell survival

Cells were seeded at a density of 1.3 × 10^5 ^cells per well in a 6-well culture plate with 3 ml of media, and incubated for 2 d before treatment with the indicated agents as described above. After 6 h, treatment medium was replaced with 3 ml of conditioned medium, and the cells were further incubated at 37°C under 5% CO for the indicated times before harvesting for the survival determination. For each agent and each time point, three wells were seeded to yield data in triplicate. Cell survival was determined by counting the number of trypan blue-negative cells. Briefly, cells were harvested by washing the culture flasks with phosphate buffered saline (Hyclone, Logan, UT) before addition of 0.5 ml trypsin (Cellgro, Hendon, VA) and incubation at 37°C for 10 min. An equal volume of Dulbecco's Eagle Modified Medium was then added, and the cells were kept at room temperature during trypan blue addition and counting. A 25 μl aliquot from the suspended cells was taken and incubated with an equal volume of trypan blue for 1 min prior to counting in the hemocytometer. Viable cells (trypan blue negative) were counted immediately after the treatment, time 0 h, at 8 h and 24 h after treatment. Percentage of viable cells is relative to 100% at time 0 h in untreated controls.

### Cloning of HO-1 cDNA

In parallel studies, the full-length mouse cDNA was obtained and subcloned downstream of a tetracycline-responsive promoter. Briefly, 500 ng of total RNA from NO-treated NIH3T3 cells was used in a first strand synthesis reaction, using commercially available reagents (Invitrogen; Carlsbad, CA). One tenth of this reaction was used as a template in a polymerase chain reaction with primers m3 (5'-ccgggatccacggtctccagtcgc-3') and m4 (5'-acgcgtcgacgttagataatgcc-3'). Amplified mHO-1 was digested with Sal1-BamH1 and subcloned into these sites into the pTREA^- ^vector. Plasmid pTREA^- ^is a derivative of commercially available pTRE (Clontech; Palo Alto, CA), in which the BGH polyadenylation signal has been removed by Hpa1-BsaA1 digestion and re-ligation.

### RNA isolation and analysis

Total RNA was extracted from the frozen samples using a commercially available kit, according to the manufacturer's instructions (RNAeasy; Qiagen, Valencia, CA). RNA concentrations were measured by UV spectroscopy at 260 nm. For analysis, 5 μg of total RNA samples were electrophoresed on denaturing agarose gels, and the separated RNA was transferred to a Nytran nylon membrane by vertical capillary transfer. Membranes were UV-crosslinked and hybridized at 68°C in Quickhybe solution (Stratagene; La Jolla, CA) to a radiolabelled probe. To probe for the full-length mHO-1 cDNA, a 1.6-kb BamH1-Sal1 fragment from plasmid pTREA^-^mHO was used. For the deadenylation assays, we used a probe corresponding to the 3' end of the mHO-1 cDNA. This probe corresponds to the 466-bp of 3' end of the mouse HO-1 cDNA, encompassed between the HindIII sites at position 1098 of the cDNA and the multiple cloning site of pTREA^-^mHO vector. After stripping, the blots were reprobed with a 1.3-kb Pst1 fragment from the human glycerlaldehyde-3-phosphate dehydrogenase (GAPDH) to control for differences in loading. These probes were labeled by the random priming method, using a commercially available system, as described by the manufacturer (Invitrogen; Carlsbad, CA). After probing, the membrane was subjected to two low-stringency washes and a high-stringency wash as suggested by the manufacturer of the hybridization solution. All blots were quantified with a Storm840 Phosphorimager (Applied Biosystems; Piscataway, NJ).

### HO-1 mRNA half-life determination

After subtraction of background signals (due to probing for the loading control, GAPDH) present at 4 h in the control samples, the HO-1 mRNA decay constants were calculated by applying a linear least squares fit to the following equation, which is a linearized version of the expected mRNA decay function:

ln(N_t_) = ln(N_0_) - λ*t*

where ln indicates the natural logarithm; N_t _is the amount of HO-1 mRNA at a given time of actinomycin treatment; N_0 _is the amount of HO-1 mRNA at time zero; λ is the decay rate per unit of time; and *t *indicates the actinomycin incubation time. The calculated slope from the linear least squares fit is the decay rate, which can be used to compute the half-lives by calculating:

t_1/2 _= ln2/λ

The calculated decay rate was also used to plot the fitted decay rate lines, shown in Figures 1B, 1D, 2B, 3B, 4C, 5C, 6C. The statistical analysis of the HO-1 mRNA half-lives reflects the difference in the slopes of the decay lines, rather than comparing the values at each time point.

### Primer extension assays

NIH3T3 cells were treated as controls (incubated with conditioned medium) or treated with 0.5 mM SPER/NO for 1 h prior to RNA extraction. Primer extension was done using 10 μg of total RNA as template and primer m6 (5'-gggcatgctgtcgggctgtgg-3'), complementary to the mouse HO-1 cDNA sequence from positions 137 to 158 nt. Primer m6 was 5' end-labeled using T4-kinase, in a 15 μl reaction containing 3.8 pmol of primer, 3.3 pmol γ-P^32 ^ATP and 2 U T4-kinase (New England Biolabs; Beverly, MA). The reaction mixture was incubated for 1 h at 37°C before gel filtration purification. Primer extension reactions were conducted using avian myoblastosis virus (AMV) – reverse transcriptase as described by the manufacturer (Promega; Madison, WI). Reaction products were separated by electrophoresis in a 5% polyacrylamide sequencing gel.

### Deadenylation assays

Deadenylation assays were conducted as described by Shyu et al. [28]. Briefly, an oligo-directed cleavage reaction was set up by adding 1 mM EDTA, and 37.5 mM primer m10 (5'ccacattggacagagttcacagc-3') to 15–25 μg samples of total RNA from each time point in a 10 μl volume. After an initial 65°C denaturation, annealing of primer m10 to its complementary sequence in HO-1 mRNA was done by slowly cooling the sample to room temperature for 15 min. RNA in this oligo-RNA hybrid was cleavaged by addition of 2.8 U of RNAse H, 20 U RNAsin and 10 U of SuperaseIn in 20 μl of a reaction buffer containing: 20 mM Hepes pH 7.6, 5 mM MgCl_2_, 30 mM KCl and 0.5 mM dithiothriol. Reactions were incubated 40 min at 37°C. RNA was precipitated by adding 20 μl NH_4_OAc, 112 μl ethanol and 25 μg glycogen as carrier and incubating this reaction at -20°C overnight. RNA was washed twice with 75% ethanol, and cleavage products were separated by denaturing electrophoresis in a 2.56% agarose gel. After electrophoresis, RNA was transferred to a Nytran nylon membrane and analyzed by northern blotting, as described before. A fraction of the total RNA was left undigested and used to measure the half-life of HO-1 mRNA, as described above. To determine the deadenylation rate, the length of the poly(A) tail attached to the 3'end fragment of HO-1 mRNA was estimated by comparison against size markers, including a fully deadenylated HO-1 fragment, made by RNase H and oligo(dT). In this analysis we considered the upper bound of the mRNA as the longest poly(A) tails in each time point, and used it for quantification. The upper boundary was determined by plotting the distribution of the hybridization signal for each 3'end product in a Storm840 Phosphorimager (Applied Biosystems; Piscataway, NJ), and by marking the area under the curve at a position in which 95–98% of the signal remained below the mark.

## List of abbreviations

AD Actinomycin D

AREs AU-rich sequence elements

DETA/NO DETA NONOate: (Z)-1 [2-(2-aminoethyl)-N-(2-ammonioethyl)amino]diazen-1-ium-1,2-diolate

HO-1 Heme oxygenase-1

MMS Methyl methane sulfonate

NMD Nonsense-mediated decay

NO Nitric oxide

UTR mRNA untranslated region

SPER/NO Spermine diazeniumdiolate: (Z)-1-[N-(3-ammoniopropyl)-N-[4-3-aminopropylammonio)butyl]-amino]diazen-1-ium-1,2-diolate

## Authors' contributions

VL carried out the laboratory experiments described in this paper, and drafted the manuscript. BD mentored this work, and participated in its design and coordination, and final revisions of the manuscript. Both authors read and approved the final manuscript.

## Supplementary Material

Additional file 1 **HO-1 mRNA stabilization and loss of cell proliferation in response to GSNO**. **A**. NIH3T3 cells were treated with increasing concentrations of GSNO for 6 h, followed by the addition of AD and monitoring the concentrations of HO-1 mRNA at the indicated times. The graph shows the fitted decay lines calculated from two independent experiments. Data points show the mean and standard errors. **B**. The same doses as for panel A were used to determine the effect of GSNO on cell viability, expressed as percentage of trypan blue-negative cells, compared to 100% at time 0 h for untreated controls. The graph shows the mean and standard error of two independent experiments. Click here for file

Additional file 2 **Mapping the transcription start site of HO-1**. **A**. NIH3T3 cells were treated as controls or with 0.5 mM SPER/NO for 1 h. Total RNA was extracted and used as a template for a primer extension reaction using primer m6, as described in the Methods section. Products were separated by denaturing polyacrylamide electrophoresis and detected by northern blotting. The site of transcription initiation is indicated by an arrow. Chain termination DNA sequencing reactions with primer m6 were electrophoresed in parallel with the primer extension products to identify their size (left 4 lanes). **B**. The transcript sequence corresponds to the brackets in panel A. The transcription start site is indicated with an asterisk (*). Click here for file

Additional file 3 **Recovery of polyadenylated (A_>30_) HO-1 mRNA from NO-treated cells**. **A**. Total RNA samples (10 μg) were subjected to RNase H treatment in the absence or presence of oligo(dT), which generates fully deadenylated HO-1 mRNA. RNA from these reactions was affinity purified on oligo(dT) Sepharose from the MicroPoly(A)Purist kit (Ambion; Austin, TX). Polyadenylated and deadenylated samples were recovered before and after purification, and products were analyzed by northern blotting. **B**. NIH3T3 cells were treated as controls or with SPER/NO for 1 h. Total RNA (40 μg from controls and 10 μg from NO-treated samples) was affinity purified using oligo(dT) Sepharose. Recovered samples were analyzed by northern blotting using the full-length HO-1 cDNA as a probe, or a GAPDH probe to normalize for loading differences. Results are representative of two independent experiments. Click here for file
