# Supplementary figures and images for: Regulation of heme oxygenase-1 mRNA deadenylation and turnover in NIH3T3 cells by nitrosative or alkylation stress
Source: BMC Mol Biol. 2007 Dec 20;8:116. doi: 10.1186/1471-2199-8-116 (PMC2246143; doi:10.1186/1471-2199-8-116)

## Slide 1
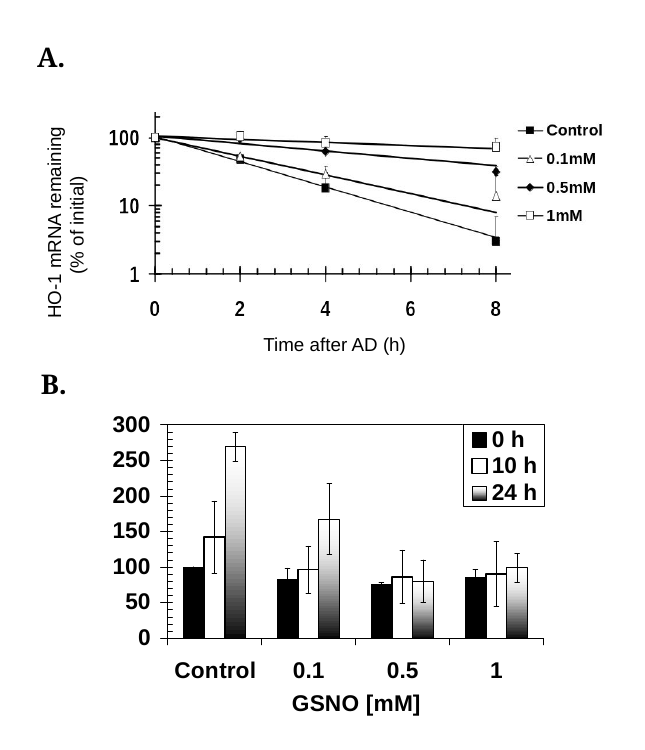

A.
 HO-1 mRNA remaining
(% of initial)
Time after AD (h)
B.

Supplement: Additional file 1 — HO-1 mRNA stabilization and loss of cell proliferation in response to GSNO. A. NIH3T3 cells were treated with increasing concentrations of GSNO for 6 h, followed by the addition of AD and monitoring the concentrations of HO-1 mRNA at the indicated times. The graph shows the fitted decay lines calculated from two independent experiments. Data points show the mean and standard errors. B. The same doses as for panel A were used to determine the effect of GSNO on cell viability, expressed as percentage of trypan blue-negative cells, compared to 100% at time 0 h for untreated controls. The graph shows the mean and standard error of two independent experiments. [file 1471-2199-8-116-S1.PPT]
