# Supplementary material for: Regulation of heme oxygenase-1 mRNA deadenylation and turnover in NIH3T3 cells by nitrosative or alkylation stress
Source: BMC Mol Biol. 2007 Dec 20;8:116. doi: 10.1186/1471-2199-8-116 (PMC2246143; doi:10.1186/1471-2199-8-116)
Supplement: Additional file 2 — Mapping the transcription start site of HO-1. A. NIH3T3 cells were treated as controls or with 0.5 mM SPER/NO for 1 h. Total RNA was extracted and used as a template for a primer extension reaction using primer m6, as described in the Methods section. Products were separated by denaturing polyacrylamide electrophoresis and detected by northern blotting. The site of transcription initiation is indicated by an arrow. Chain termination DNA sequencing reactions with primer m6 were electrophoresed in parallel with the primer extension products to identify their size (left 4 lanes). B. The transcript sequence corresponds to the brackets in panel A. The transcription start site is indicated with an asterisk (*). [file 1471-2199-8-116-S2.PPT]

## Slide 1
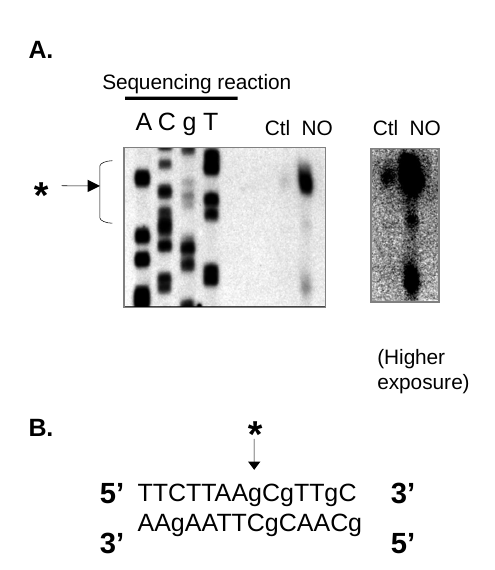

A.
Sequencing reaction
 A C g T
Ctl NO
Ctl NO
 *
(Higher
exposure)
 *
B.
5’
3’
TTCTTAAgCgTTgC
AAgAATTCgCAACg
3’
5’
