# Supplementary material for: Regulation of heme oxygenase-1 mRNA deadenylation and turnover in NIH3T3 cells by nitrosative or alkylation stress
Source: BMC Mol Biol. 2007 Dec 20;8:116. doi: 10.1186/1471-2199-8-116 (PMC2246143; doi:10.1186/1471-2199-8-116)
Supplement: Additional file 3 — Recovery of polyadenylated (A>30) HO-1 mRNA from NO-treated cells. A. Total RNA samples (10 μg) were subjected to RNase H treatment in the absence or presence of oligo(dT), which generates fully deadenylated HO-1 mRNA. RNA from these reactions was affinity purified on oligo(dT) Sepharose from the MicroPoly(A)Purist kit (Ambion; Austin, TX). Polyadenylated and deadenylated samples were recovered before and after purification, and products were analyzed by northern blotting. B. NIH3T3 cells were treated as controls or with SPER/NO for 1 h. Total RNA (40 μg from controls and 10 μg from NO-treated samples) was affinity purified using oligo(dT) Sepharose. Recovered samples were analyzed by northern blotting using the full-length HO-1 cDNA as a probe, or a GAPDH probe to normalize for loading differences. Results are representative of two independent experiments. [file 1471-2199-8-116-S3.PPT]

## Slide 1
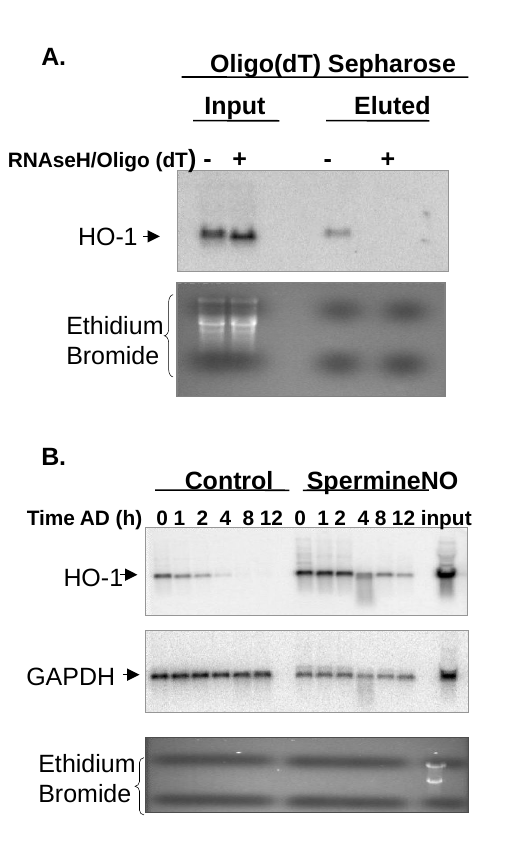

A.
Oligo(dT) Sepharose
Input
Eluted
 RNAseH/Oligo (dT) - + - +
HO-1
Ethidium
Bromide
B.
SpermineNO
Control
Time AD (h)
 0 1 2 4 8 12 0 1 2 4 8 12 input
HO-1
GAPDH
Ethidium
Bromide
